# Cyanoacrylate in Colorectal Surgery: Is It Safe?

**DOI:** 10.3390/jcm12155152

**Published:** 2023-08-07

**Authors:** Anna D’Amore, Pietro Anoldo, Michele Manigrasso, Giovanni Aprea, Giovanni Domenico De Palma, Marco Milone

**Affiliations:** 1 Department of Clinical Medicine and Surgery, “Federico II” University of Naples, 80131 Naples, Italy; michele.manigrasso@unina.it (M.M.); giovanni.aprea@unina.it (G.A.); giovannidomenico.depalma@unina.it (G.D.D.P.); milone.marco.md@gmail.com (M.M.); 2 Department of Advanced Biomedical Sciences, “Federico II” University of Naples, 80131 Naples, Italy; pietro.anoldo@gmail.com

**Keywords:** cyanoacrylate, colorectal surgery, minimally invasive surgery, anastomosis, leakage, inflammation, adhesions

## Abstract

Anastomotic leakage (AL) of a gastrointestinal (GI) anastomosis continues to be an important complication in GI surgery. Since its introduction more than 60 years ago, Cyanoacrylate (CA) has gained popularity in colorectal surgery to provide “prophylaxis” against AL. However, although in surgical practice it is increasingly used, evidence on humans is still lacking. The aim of this study is to analyze in humans the safety of CA to seal colorectal anastomosis. All consecutive patients from Jannuary 2022 through December 2022 who underwent minimally invasive colorectal surgery were retrospectively analyzed from a prospectively maintained database. Inclusion criteria were a histological diagnosis of cancer, a totally minimally invasive procedure, and the absence of intraoperative complications. 103 patients were included in the study; N-butyl cyanoacrylate with metacryloxisulfolane (Glubran 2^®^) was used to seal colorectal anastomosis, no adverse reactions to CA or postoperative complications related to inflammation and adhesions occurred; and only one case of AL (0.9%) was recorded. We can consider this study an important proof of concept on the safety of CA to seal colorectal anastomosis. It opens the possibility of starting prospective and comparative studies in humans to evaluate the effectiveness of CA in preventing colorectal AL.

## 1. Background

Anastomotic leakage (AL) of a gastrointestinal (GI) anastomosis continues to be an important complication in GI surgery. The development of AL depends on several risk factors, which can be divided into patient-related risk factors and operative factors.

Recently, the adoption of surgical innovations, which include stapling techniques, intraoperative air testing, direct sigmoidoscopic visualization, and the use of tissue adhesives to seal colorectal anastomoses, has reduced the AL rate from 3% to 23% [1,2,3,4,5,6,7,8,9,10,11,12,13,14,15]. Tissue adhesives are less invasive than sutures and staples, and they do not affect the wound-healing process due to their flexibility. Moreover, their application is easy and standardizable, resulting in less variation between different surgeons. There are different categories of tissue adhesives based on their chemical composition: fibrin sealants, albumin-based compounds, cyanoacrylates, hydrogels and collagen compounds.

Cyanoacrylate (CA) is a type of tissue adhesive that is CE-certified for internal and external use. It has haemostatic, adhesive and antiseptic properties and, once injected, it polymerizes in contact with vital tissues to create an elastic layer with a high tensile strength. Since its introduction more than 60 years ago, the use of CA has increased in various fields of surgical practice, such as cardiac surgery, pediatric cardiac and general surgery, vascular surgery, neurosurgery, ophthalmology, maxillofacial surgery, odontostomotology, plastic surgery, thoracic surgery, gyneacological and breast surgery, urological surgery, digestive endoscopy, interventional radiology, vascular neuroradiology, interventional cardiology and angioplasty. Particularly, it has gained popularity in GI surgery [16,17] for different purposes, such as to seal gastrointestinal and rectal anastomoses, anastomoses in the biliary tract and appendix stumps, to control hemostasis, to close mesenteric defects and to treat gastrocutaneous, anal and perianal fistulas. In this study, we focused on its role as a sealant in colonic anastomosis. Other applications of CA, including its use in emergency surgical procedures and in the treatment of upper and lower gastrointestinal fistulas, are very interesting. In 1998, Dermabond (2-octylcyanoacrylate) was approved for topical skin wound closure by the Food and Drug Administration (FDA) [18]. Since the medical use of CA tissue adhesives has been established, many experimental animal studies have been performed to evaluate their use in colonic anastomosis [19,20]. However, although in surgical practice CA is increasingly used, evidence on humans is still lacking. This study involved cancer patients who underwent minimally invasive colorectal surgery. In all operations, N-butyl cyanoacrylate with metacryloxisulfolane (Glubran 2^®^), CE certified for internal and external use, was used as an anastomotic sealant and it was applied to colonic anastomosis after its creation with a Spray Device for Surgical Glue Glubran 2. N-butyl cyanoacrylate with metacryloxisulfolane (Glubran 2^®^) was also indicated as CA in the rest of the text. The aim of this study was to analyze in humans the safety of CA for colorectal anastomosis.

## 2. Materials and Methods

All consecutive patients from Jannuary 2022 through December 2022 who underwent minimally invasive colorectal surgery were retrospectively analyzed from a prospectively maintained database. Inclusion criteria were histological diagnosis of cancer, a totally minimally invasive procedure and the absence of intraoperative complications to avoid bias in the safety assessment of CA to seal colorectal anastomosis. Written informed consent to undergo surgery and to use their medical records for scientific purposes was obtained from all subjects enrolled in the study after hospitalization.

Before surgery, each patient received a complete history and physical examination with blood tests and tumoral marker research, a colonoscopy with both a biopsy of the lesion to obtain a histological diagnosis of cancer and endoscopic tattooing of the lesion to facilitate the surgeon’s work, a total body Computed Tomography (CT) for tumor staging and a Magnetic Resonance Imaging (MRI) in the case of medium-low rectal lesions. After a careful evaluation of the clinical case, the operative technique was chosen. Moreover, after the patient’s admission, bowel preparation with an osmotic laxative, when it was possible and preoperative antibiotic and heparin prophylaxis were administered to each patient.

All patients underwent minimally invasive procedures under general anesthesia. All operations were performed by experienced colorectal surgeons. In a right colectomy, the ileocolic pedicle was identified and the peritoneum of the mesentery was opened, creating a mesenteric window. After identification and preservation of the right ureter, duodenum and pancreatic head, Toldt’s fascia was separated from Gerota’s plane. The ileocolic pedicles, the right colic vessels and the right branch of the middle colic vessels were tied at their origin, and the right colon was mobilized from the right parietocolic gutter. Then the right hemicolectomy with a linear stapler and the subsequent intracorporeal ileo-colic anastomosis fashioning in a side-to-side isoperistaltic way were performed. In the left colectomy, the surgeon started with the colo-epiploic detachment and the mobilization of the splenic flexure. When Toldt’s fascia was separated from Gerota’s plane, preserving the left ureter and gonadic vessels, the Inferior Mesenteric Vein (IMV) and the Inferior Mesenteric Artery (IMA) could be isolated, clipped, and divided. A left hemicolectomy with a linear stapler and the subsequent colorectal end-to-end anastomosis according to the Knight–Griffen technique were performed. In the anterior rectal resection, after the aforementioned steps, a Partial or Total Mesorectal Excision (PME or TME) was added to the procedure. In the case of segmental splenic flexure resection, after the descending and transverse colons were mobilized, the left branches of the middle colic vessels and the left colic artery were isolated, clipped, and ligated. For transverse colon resection, after the complete mobilization of both colic flexures, a resection of the mesentery, including the middle colic artery, was performed. Segmental resections included intracorporeal colo-colic anastomosis fashioning in a side-to-side isoperistaltic way. Moreover, to ensure that colorectal vascularization was adequate, the Near-Infrared Fluorescence-Indocyanine Green (NIR/ICG) system was carried out according to a standardized technique for each patient at two different moments: before and after the anastomosis creation to control the proper stump vascularization and the anastomosis perfusion, respectively. In detail, a bolus of 0.2 mg/kg of ICG was administered by the abesthesiologist before the intestinal resection and after the anastomosis creation, and if adequate vascularization was present, it was visible after 25 s.

After the creation of the colonic anastomosis and control of its proper vascularization with the ICG-test, one milliliter of N-butyl cyanoacrylate with metacryloxisulfolane (Glubran 2^®^) (CA) was applied to the colonic anastomosis by using a Spray Device for Surgical Glue Glubran 2. This device allows you to apply CA in nebulized form and to evenly release it on the area of interest in 60–90 s. After its application, the surgeon placed a gauze over or in contact with CA, paying attention to removing the excess product. If the intervention was performed with the robotic approach, CA was applied by an assistant surgeon.

During the postoperative course, patients were evaluated with clinical monitoring and daily blood tests to control and quickly identify the occurrence of possible complications. After discharge, the patients were submitted to a check after 7, 30, 60, and 90 days.

The study was conducted according to the guidelines of the 1975 Declaration of Helsinki and the protocol was approved by the Ethics Committee of Federico II University of Naples (331/18).

The study findings have been reported in compliance with the STROBE checklist [21].

Demographic information and surgery-related data were extracted. Demographic information included sex, age, BMI, obesity, smoking, comorbidities, ASA score and previous abdominal surgery. Surgery-related data involved operative time, time to first flatus and stool, time to tolerance to a solid diet, length of hospital stay and postoperative complications (adverse reactions to CA, AL and other complications). Complications were classified according to the Clavien–Dindo Score.

The primary outcome was the occurrence of adverse reactions to CA, such as inflammation, adhesions, allergic reaction and anaphylaxis. Particularly, inflammation was defined as the increase in inflammatory markers with the presence of fever. In detail, the inflammation markers used are white blood cell counts and C-Reactive Protein (CRP). The reference range of our analysis laboratory was used, and it was 4.5–11.0 × 10^3^/μL for white blood cell counts and 0–5.0 mg/L for C-Reactive Protein. There was an increase in inflammatory marker when the markers value exceeded the highest value of the range.

Adhesions were defined as the presence of nausea, abdominal pain, and extended time to first flatus and stool, which required a deeper analysis with Computed Tomography (CT) and reintervention. CT evaluated the presence of adhesions, which were confirmed during reintervention.

Secondary outcomes were the occurrence of AL, other complications, and postoperative recovery assessment. Anastomotic leakage is defined as a dehiscence of the intestinal wall at the anastomotic site; it was suspected when fever, abdominal pain and fecal matter in abdominal drainage occurred. At computed tomography, it was characterized by an abscess and gas around the anastomotic site and by the presence of communication between inside and outside the intestinal tract at the contrast enema. Anastomotic leakage was considered a complication when it required a surgical re-intervention.

The SPSS 28 system (SPSS Inc., Chicago, IL, USA) was used to perform statistical analysis. Continuous data were expressed as mean ± standard deviation; categorical variables were expressed as percentages.

## 3. Results

156 patients were eligible for study inclusion, and they were enrolled in the analysis, but 53 were excluded because of the following reasons: 22 patients underwent surgery for diverticular disease; 20 patients received an open approach; eight patients required conversion to open because of adhesive syndrome and three patients had intraoperative complications, including two intraoperative bleedings and one splenic lesion.

Thus, 103 patients were included in the study. 57 patients previously underwent open or minimally invasive abdominal surgery, while 46 of them did not receive previous abdominal interventions, and 14 patients underwent neoadjuvant therapy.

Demographic characteristics, tumor localization, and type of surgical procedure are shown in Table 1.

In addition, we also analyzed demographic characteristics, dividing the patients according to the surgical procedures. Of the 33 patients who underwent right colectomy, 19 (57.6%) were men and 14 (42.4%) were women; the mean age was 69.3 ± 10.2 and the mean BMI was 25.5 ± 3.15. 14 patients (42.4%) presented an ASA grade 2, 17 (51.5%) of them an ASA grade 3, and the other two (6.1%) an ASA grade 4. Arterial hypertension affected 24 patients (72.7%), diabetes affected eight patients (24.2%), obese patients were five (15.15%), and smokers were 11 (33.3%). Of the two patients who underwent transverse colon resection, one (50%) was a man and one (50%) was a woman; the mean age was 67.5 ± 9.2 and the mean BMI was 23.5 ± 0.7; both patients (100%) presented an ASA grade 3; arterial hypertension affected one patient (50%); patients were not obese; and one of them was a smoker (50%). Of the six patients who underwent splenic flexure resection, three (50%) were men and three (50%) were women; the mean age was 67.6 ± 8.6 and the mean BMI was 26.2 ± 7.5; four patients (66.7%) presented an ASA grade 2 and two (33.3%) of them an ASA grade 3; arterial hypertension affected two patients (33.3%); patients were not obese; and two of them were smokers (33.3%). Of the 31 patients who underwent left colectomy, 21 (67.7%) were men and 10 (32.3%) were women; the mean age was 66.1 ± 10.6 and mean BMI was 26.9 ± 5.9, 18 patients (58.1%) presented an ASA grade 2 and 13 (41.9%) of them an ASA grade 3, arterial hypertension affected 23 patients (74.2%), diabetes affected seven patients (22.6%); obese patients were eight (25.8%); and smokers were six (19.3%). Of the 27 patients who underwent anterior resection of the rectum, 14 (51.9%) were men and 13 (48.1%) were women; the mean age was 64.6 ± 9.5 and the mean BMI was 26.1 ± 5.7; 14 patients (51.8%) presented an ASA grade 2, 11 (40.7%) of them an ASA grade 3, and the other two (7.4%) an ASA grade 4, arterial hypertension affected 17 patients (63%), diabetes affected three patients (11.1%), obese patients were four (14.8%), and smokers were six (22.2%). Of the other four patients, two underwent total colectomy, one underwent subtotal colectomy, and another underwent proctocolectomy; one (25%) were men and three (75%) were women; the mean age was 64.25 ± 9.9 and the mean BMI was 26.75 ± 3.3; 1 patient (25%) presented an ASA grade 2, two (50%) of them an ASA grade 3, and another (25%) an ASA grade 4; arterial hypertension affected one patient (25%); one patient was obese (25%).

All patients underwent minimally invasive procedures, with the laparoscopic approach in 61 cases and the robotic approach in 42 cases using CA to seal the anastomosis. In five patients (4.8%), the planned site of resection was changed intraoperatively due to inadequate perfusion at the NIR/ICG system, and the anastomosis was properly vascularized.

Surgery-related data are shown in Table 2.

No adverse reactions to CA or complications related to inflammation and adhesions occurred; only one case of AL (0.9%) was recorded in a patient with a BMI greater than 30 kg/m^2^. Other complications were classified according to the Clavien–Dindo Score. Particularly, a Clavien–Dindo grade 1 occurred in 8 patients (8%); 6 of them had nausea (6%) and were treated with antiemetic drugs, while the other two patients had fever (2%) and were treated with antipyretic drugs. Clavien–Dindo grade 2 occurred in 11 patients who had anemia (11%) and who received blood transfusions. In the details of patients with nausea, two underwent right colectomy, two underwent left colectomy, and the other two underwent anterior resection of the rectum. Of the patients with fever, one underwent a right colectomy and the other underwent a left colectomy. Of the patients with anemia, three underwent right colectomy, three underwent left colectomy, four underwent anterior resection of the rectum and one underwent total colectomy.

Other postoperative complications are shown in Table 3.

The mean operative time was 210.2 ± 55.21. The mean length of hospital stay was 6.08 ± 2.63, the mean time to first flatus was 1.91 ± 0.87, the mean time to first stool was 3.01 ± 1.72 and the mean time to tolerance to a solid diet was 3.27 ± 1.44. Even in this case, surgery-related dates were analyzed, dividing the patients according to the surgical procedures. In details, of the 33 patients who underwent right colectomy, 22 (66.7%) underwent laparoscopic procedures and 11 (33.3%) underwent robotic procedures, with a mean time to first flatus of 2 ± 0.9, a mean time to first stool of 3.7 ± 1.9, a mean length of hospital stay of 5.8 ± 2.7 and a mean time to tolerance to solid diet of 3.5 ± 1.7. The two transverse colon resections were robotic procedures (100%) with a mean time to first flatus of 2.5 ± 0.7, a mean time to first stool of 4.5 ± 0.7, a mean length of hospital stay of 10 ± 5.7 and a mean time to tolerance to a solid diet of 4 ± 1.4. Of the six patients who underwent splenic flexure resection, five (83.3%) underwent laparoscopic procedures and one (16.7%) underwent robotic procedures with a mean time to first flatus of 2.3 ± 0.5, a mean time to first stool of 2.8 ± 0.4, a mean length of hospital stay of 5.7 ± 1.5 and a mean time to tolerance to solid diet of 3 ± 0. Of the 31 patients who underwent left colectomy, 15 (48.4%) underwent laparoscopic procedures and 16 (51.6%) underwent robotic procedures with a mean time to first flatus of 1.9 ± 1.04, a mean time to first stool of 3.1 ± 1.5, a mean length of hospital stay of 5.9 ± 2.4 and a mean time to tolerance to solid diet of 3.3 ± 1.2. Of the 27 patients who underwent anterior resection of the rectum, 16 (59.3%) underwent laparoscopic procedures and 11 (40.7%) underwent robotic procedures, with a mean time to first flatus of 1.6 ± 0.64, a mean time to first stool of 2.4 ± 1.9, a mean length of hospital stay of 6.1 ± 2.8 and a mean time to tolerance to solid diet of 3 ± 1.6. The other four patients, of whom two underwent total colectomy, one underwent subtotal colectomy, and another underwent proctocolectomy, all (100%) underwent laparoscopic procedures with a mean time to first flatus of 2.75 ± 0.5, a mean time to first stool of 3.75 ± 1.3, a mean length of hospital stay of 8.5 ± 4.5 and a mean time to tolerance to solid diet of 3.75 ± 1.

No complications were recorded when patients were submitted to a check after 7, 30, 60 and 90 days.

## 4. Discussion

Anastomotic leakage (AL) is a serious complication in colorectal surgery. In the past, its incidence ranged from 17% to 77% [22,23,24,25,26]. It is a multifactorial problem. The development of AL depends on several risk factors that vary between different populations and can be divided into patient-related risk factors and operative factors. In detail, patient-related risk factors involve comorbidity, body mass index and drug use, while operative factors include the surgeon’s experience, after-hours surgery, anastomotic location and operating time [13,14,15]. Of interest, one of the causes of AL is ischemia, among the comorbidities, it is important to consider vascular diseases such as atherosclerosis, whose risk factors are hypertension, dyslipidemia, smoking, and diabetes mellitus [15]. About BMI, it has been shown that a BMI ≥ 30 is predictive for AL [27]. About drug use, it has been reported that patients on corticosteroids who are in poor clinical conditions, who suffer major blood loss or when the intervention is longer than usual could have a higher risk of AL [28]. A surgeon’s experience is very important in influencing outcomes following colorectal cancer surgery, especially rectal cancer surgery; in fact, this type of surgery should be performed by high-volume surgeons [29]. Moreover, the creation of the anastomosis is one of the most technically difficult steps, particularly when it involves the rectum with an AL risk that is seven times higher when it is located to the right colon and four times higher when it is located to the left colon [30]. However, even in high-volume centers there are several features that influence AL development, such as delays in diagnosis or neoadjuvant therapy. After-hours surgery is defined as the period in which patients undergo an intervention performed by an on-call operating team and it has been reported that these patients have more than a twofold increased risk of AL [15]. Finally, prolonged operative time could lead to AL, probably because it could reflect intraoperative complications [30].

Recently, AL rates in colorectal surgery (less than 3–23%) and the risk of a serious clinical leakage [31] have decreased due to several surgical innovations, which include stapling techniques, intraoperative air testing, direct sigmoidoscopic visualization and the use of tissue adhesives to seal colorectal anastomosis [1,2,3,4,5,6,7,8,9,10,11,12,13,14,15]. Tissue adhesive can be defined as any substance that allows for polymerization both to hold tissues together and to avoid leakage [32]. They are minimally invasive and their application is easy and standardizable.

N-butyl cyanoacrylate with metacryloxisulfolane (CA) is a class III medical device for internal and external use. It is a synthetic cyanoacrylate liquid modified by the addition of a monomer. It has haemostatic and adhesive properties; moreover, once solidified, it provides an effective antiseptic barrier against infectious agents or pathogens in surgical settings. It is a waterproof, pale yellow, transparent liquid that polymerizes in contact with vital tissues to create an elastic layer with a high tensile strength, ensuring firm adhesion to tissue. A temperature of approximately 45 °C is generated during the polymerization reaction. The layer fits the anatomy of the underlying tissue. The polymerization time depends on the type of material with which CA comes into contact. After its application, the glue starts to set after 1–2 s and ends its setting reaction in 60–90 s. Once set, tissue or surgical gauze may be placed over or in contact with CA.

There are several accessory devices for CA administration, such as an insulin syringe, drop control device, dispensing tip, laparoscopic catheter, spray device that we used for our cases, Glubran 2 sealing device and glutack.

Since its introduction more than 60 years ago, CA has gained popularity in colorectal surgery [16,17,33] showing lower toxicity to the tissues. However, it is used in various fields of surgical practice, especially in GI surgery, for different purposes, such as to seal gastrointestinal and rectal anastomoses, anastomoses in the biliary tract and appendix stumps, to control hemostasis, to close mesenteric defects and to treat gastrocutaneous, anal and perianal fistulas. In this study, we focused on its role as a sealant in colonic anastomosis, and the evidence we found in the literature is controversial [34,35,36,37,38,39,40,41,42,43,44,45,46,47,48]. On a clinical point of view, some surgeons believe in the useful advantages of CA in preventing AL. Other surgeons think about the potential risks of CA in creating inflammation and adhesions, which could lead to possible postoperative complications. So far, the only certain adverse effects could be rare inflammatory reactions at the application site due to an excessive amount of CA, allergic reaction and anaphylaxis.

This is the first study to evaluate the safety of a CA to seal colorectal anastomosis in humans. In fact, available data on the use of CA both for sutureless colonic anastomosis and for colorectal anastomotic sealant were only shown in experimental animal studies [34,35,36,37,38,39,40,41,42,43,44,45,46,47,48]. Bae et al. [36] performed a study on male Sprague-Dawley rats and divided them in three groups: group 1 received an anastomosis sutured in a single layer; group 2 received an anastomosis fixed using CA and group 3 received an anastomosis both sutured and sealed with CA. They did not observe AL in any group, showing that CA could be a useful technique for sutureless colonic anastomosis. Similar results were reported by Kanellos et al. [37]. Of interest were several studies on pigs [19,42,48,49,50,51] which had physiological reactions similar to those in humans. These studies demonstrated some advantages of CA. In detail, Tebala et al. performed a study on Wistar rats and Landrace pigs [48] to analyze the tissue reaction to CA and its adhesive features and another study on Landrace pigs [19] to evaluate the efficacy and patient tolerance of CA when employed as a sealant for high-risk intestinal anastomoses. It was found that CA had a good adhesive effect in the first study [48], that it was efficacious as a sealant for high-risk anastomoses and that it supported the wound healing process in the second study [19]. Similar results were also reported by Wu et al. [49], who demonstrated that CA is the most important factor to determine the strength of both normal and insufficiently sealed colorectal anastomoses and that the mechanical strength of a colorectal anastomosis increased with CA application, probably contributing to the decrease in AL occurrence. Moreover, Boersema et al. [50], investigating the effect of CA in the prevention of leakage in a porcine model of ischemic colorectal AL, found that its use prevented from leakage in cases with partially ischemic colo-colonic anastomosis. Paral et al. [51] compared the resistance of glued versus stapled colonic anastomosis to intraluminal pressures at different times during healing. They found that not only gluted anastomoses resisted pressure significantly higher than physiological pressures, but also that CA did not affect anastomose healing. On the other hand, in a clean, contaminated or bacterial peritonitis environment [52], CA determined inflammatory reaction, necrosis and adhesion formation.

Of interest was a case report [53] of a patient who underwent an emergency surgery of a total gastrectomy with CA application on the side-to-side esophago-jejunal (E-J) anastomosis after caustic ingestion. This study showed that even in emergency surgical procedures, CA could be used to seal the anastomosis due to its utility and efficacy. This was important food for thought because it increased the fields in which CA could be use, such as non-elective interventions and operations complicated by intraoperative problems. Their application is easy and standardizable, so their use in emergency situations would not lead to a significant impediment for the surgeon, who could find CA a valid tool that is fast and convenient to use. In this study, intraoperative complications were exclusion criteria and patients affected by them were not included in the analysis to avoid bias in the safety assessment of CA to seal colorectal anastomosis. However, the aforementioned case report led us to think that intraoperative complications did not influence the judgment on CA safety but high-quality studies on humans have to be performed on this topic to obtain certain results.

Between other applications of CA, the treatment of upper and lower gastrointestinal fistulas was one of the most interesting. A few case reports were presented in the literature [54,55,56,57]. In details, Anoldo et al. [54] reported a case of cervical esophageal perforation that was successfully treated with CA injection after the failure of other conservative options. Thus, it was possible to consider CA a promising minimally invasive alternative for the treatment of cervical esophageal perforation. Alharbi et al. [55] described a case of a low-output enterocutaneus fistula treated with CA applied to sutures. They showed that the glue can be a safe, minimally invasive treatment for this type of fistula. Moreover, it seems possible to use it safely also in pediatric surgery, as shown by Hosseini et al. [56], who reported the treatment of cases of tracheoesophageal atresia with fistula, hypospadias, cases of vesicutanouse fistula after bladder extrophy and cases of cloacal extrophy.

All patients enrolled in the analysis underwent minimally invasive procedure, with laparoscopic approaches in 61 cases and robotic approaches in 42 cases. Laparoscopic surgery is the gold standard for the treatment of colorectal cancer given its proven advantages, such as smaller abdominal incisions, lower manual traction and abdominal tissue manipulation, shorter postoperative recovery with better operative outcomes and oncologic safety [57,58]. However, the laparoscopic approach presents some technical difficulties when it is performed in a small field, such as the pelvis, requiring high surgeon expertise. The robotic approach could help solve these problems with a 3D-magnified view, better ergonomics and lower physiologic tremor due to EndoWrist instruments. In fact, it was reported that the robotic technique for rectal resection is the best way to perform a complete TME [59].

In our series before CA application on colorectal anastomosis, its proper vascularization was checked with the NIR/ICG system. Indocyanine green (ICG) is a tricarbocyanine compound that, once injected intravenously, through blood perfusion reached the liver, where its fluorescence was captured and activated by a system with the power of a light emitted by an LED. ICG fluorescence decreased when the vascularization of a tissue was reduced [60].

After checking the anastomotic vascularization, it could proceed with the CA application. The amount we used was one milliliter of product in 60–90 s. The device we preferred was a Spray Device for Surgical Glue Glubran 2 to deliver CA in an atomized form evenly on the interested area. It was important not to apply more than the fixed dose because it could lead to inflammatory reactions, allergic reactions, and anaphylaxis, thus causing a possible bias in the safety assessment of CA to seal colorectal anastomosis. If an excess of product occurred, the surgeon removed it with gauze. Thus, CA dosage played a central role in its application. Unfortunately, current literature does not show certain results on this. However, a high dose of CA could cause tissue destruction and adhesion formation, so it was important to remove excess CA from the colonic anastomosis.

According to our results, we can propose the safety of CA as an anastomotic sealant. All 103 consecutively enrolled patients had no adverse reactions to CA or postoperative complications related to inflammation or adhesions. This can support the safety of CA in colorectal cancer patients and it can help reassure surgeons who worry about the potential risks of tissue adhesives and encourage them to use it as a sealant. Regarding leakage prevention, only one case of leakage occurred (0.9%) in our series, with an AL rate lower than that shown in the literature (3 to 23%). It could make us think that it is possible that the adhesive properties of CA decrease the risk of leakage development. Anyway, CA in our study certainly did not lead to an increase of leakage occurrences. However, this result should not be generalized to all colorectal cancer series due to the limitations of the study. The main one is the retrospective observational design, which can lead to potential patients’ selection bias, making it difficult to draw firm results. Another limitation is the relatively small sample size, involving only oncological patients who underwent totally minimally invasive procedures without intraoperative complications. Therefore, other comparative studies with the largest sample sizes are needed to give certain results.

We can consider this study only an important proof of concept on the safety of CA to seal colorectal anastomosis. It opens the possibility of starting prospective and comparative studies in humans to evaluate the effectiveness of CA in preventing colorectal AL and to explore its real advantages in clinical practice.

## 5. Conclusions

CA is safe as an anastomotic sealant. It is less invasive than sutures and staples. In addition, it has lower toxicity to the tissues. The application of a fixed amount of product to the colorectal anastomosis seems to avoid inflammatory reactions, allergic reactions, anaphylaxis, and complications related to inflammation or adhesions. About leakage prevention, there are promising results but high-quality studies with the widest sample sizes remain required to evaluate its effectiveness and its real advantages in humans.

## Figures and Tables

**Table 1:** Demographic characteristics, tumor localization, and type of surgical procedure of the included patients.

Characteristics	
M/F (%)	57/46 (55%/45%)
Age (years)	67.2 ± 10.1
BMI (kg/m^2^)	26.2 ± 5
ASA 1 (%)	0
ASA 2 (%)	50 (48%)
ASA 3 (%)	48 (47%)
ASA 4 (%)	5 (5%)
Hypertension (%)	72 (70%)
Diabetes (%)	18 (17%)
Obesity (%)	18 (17%)
Smoke (%)	26 (25%)
**Tumor Localization**	
Caecum (%)	5 (5%)
Right colon (%)	18 (17%)
Liver flexure (%)	7 (7%)
Transverse colon (%)	5 (5%)
Splenic flexure (%)	4 (4%)
Descending colon (%)	6 (6%)
Sigma (%)	26 (25%)
Rectum (%)	24 (23%)
Colorectal junction (%)	6 (6%)
Right colon and colorectal junction (%)	1 (1%)
Caecum and sigma (%)	1 (1%)
**Type of Surgery Procedure**	
Right colectomy (%)	33 (32%)
Transverse colon resection (%)	2 (2%)
Splenic flexure resection (%)	6 (6%)
Left colectomy (%)	31 (30%)
Anterior resection of the rectum (%)	27 (26%)
Total colectomy (%)	2 (2%)
Subtotal colectomy (%)	1 (1%)
Proctocolectomy (%)	1 (1%)

Categorical variables are expressed as numbers and (percentages), while continuous variables are expressed as mean ± SD. M: male; F = female; BMI: Body Mass Index; ASA score: American Society of Anesthesiology score.

**Table 2:** Surgery-related data of the included patients.

Surgery-Related Data	
First flatus (days)	1.91 ± 0.87
First stool (days)	3.01 ± 1.72
Lenght of hospital stay (days)	6.08 ± 2.63
Time to tolerance to solid diet (days)	3.27 ± 1.44

Categorical variables are expressed as numbers and percentages, while continuous variables are expressed as mean ± SD.

**Table 3:** Postoperative complications.

Clavien-Dindo (CD) Score	N (%)
CD 1	8 (8%)
Nausea	6 (6%)
Fever	2 (2%)
CD 2	11 (11%)
Anemia	11 (11%)
CD 3	1 (1%)
CD 4	0
CD 5	0

Categorical variables are expressed as numbers and (percentages).

## Data Availability

The data presented in this study are available on request from the corresponding author.
